# Influence of Strong Shear Field on Structure and Performance of HDPE/PA6 In Situ Microfibril Composites

**DOI:** 10.3390/polym16081032

**Published:** 2024-04-10

**Authors:** Junwen Zhang, Yiwei Zhang, Yanjiang Li, Mengna Luo, Jie Zhang

**Affiliations:** College of Polymer Science and Engineering, State Key Laboratory of Polymer Materials Engineering, Sichuan University, Chengdu 610065, China; wenjunzh11@outlook.com (J.Z.); z403084973@163.com (Y.Z.); 13881505715@163.com (Y.L.); z1907747660@163.com (M.L.)

**Keywords:** HDPE, in situ microfibril composites, strong shear field, shish-kebab, hybrid shish-kebab

## Abstract

As one of the most widely applied general-purpose plastics, high-density polyethylene (HDPE) exhibits good comprehensive performance. However, mechanical strength limits its wider application. In this work, we introduced the engineering plastic PA6 as a dispersed phase to modify the HDPE matrix and applied multiple shears generated by vibration to the polymer melt during the packing stage of injection molding. SEM, 2D-WXRD and 2D-SAXS were used to characterize the morphology and structure of the samples. The results show that under the effect of a strong shear field, the dispersed phase in the composites can form in situ microfibers and numerous high-strength shish-kebab and hybrid shish-kebab structures are formed. Additionally, the distribution of fibers and high-strength oriented structures in the composites expands to the core region with the increase in vibration times. As a result, the tensile strength, tensile modulus and surface hardness of VIM-6 can reach a high level of 66.5 MPa, 981.4 MPa and 72, respectively. Therefore, a high-performance HDPE product is successfully prepared in this study, which is of great importance for expanding the application range of HDPE products.

## 1. Introduction

High-density polyethylene (HDPE), as a high-crystallinity, non-polar thermoplastic resin, has good comprehensive properties such as low toxicity, excellent cold resistance, good mechanical performance, great chemical resistance and ease of processing [1,2,3,4,5,6]. Therefore, it has become one of the most widely applied general-purpose plastics. However, with the advancement of science and technology, the mechanical strength of existing HDPE can no longer meet the demand for high-strength plastic products [7]. Therefore, it is of great significance to further improve the mechanical properties of HDPE to expand its application range.

High-performance polymer composites have been widely researched and applied owing to their excellent mechanical properties [8,9]. Adding fibers to the polymer matrix to prepare polymer composites is one of the common methods of polymer reinforcement [10,11]. At present, the commonly used reinforcing fibers are mainly high-strength inorganic fibers, such as glass fibers (GFs) [12,13], carbon fibers (CFs) [14,15], etc. But during the process of manufacturing these composites, the fibers may break as a result of shearing and agitation by the processing equipment, which reduces the length-to-diameter (L/D) ratio of the fibers and even leads to a decrease in the mechanical properties of the material [16]. Fortunately, the in situ microfibril composite (MFC) has stood out from the traditional polymer composites and attracted the attention of many researchers due to its great potential in mechanical enhancement and huge advantage in manufacturing over the past few decades [17,18]. Compared with common inorganic fiber polymer composites, the fibers of MFCs are formed during processing, which prevents fibers breaking caused by screw agitation. Traditional methods of preparing MFCs are usually divided into three steps: melt blending, stretching into fibers, processing and molding [19,20,21]. MFCs obtained by this method usually need to undergo a secondary process after the formation of in situ microfibers, which may lead to the aggregation of fibers, thereby affecting the final properties of the products [8]. Therefore, if it is possible for the dispersed phase to form in situ fibers directly during the molding process, it will not only ensure the quality of the fibers but also greatly simplify the processing. In recent years, several studies [8,22,23,24,25,26,27] have proven the feasibility of this method. For example, Qin et al. [22] obtained high-performance HDPE/PET MFCs through volume-pulsatile injection molding (VPIM). The results demonstrated that under the effect of the vibration force field, the PET dispersed phase formed rigid microfibers and the strength as well as the toughness of the products greatly improved. The tensile strength and impact strength of the material reached 31.1 MPa and 41.4 MPa, with an increase of 29% and 658%, respectively. Jiang et al. [27] successfully prepared PP/PS in situ microfibril composites by fused filament fabrication (FFF) and observed that the tensile strength of the product increased by 43.6%. Shen et al. [24] combined in situ fiber formation with the stacked extrusion technique to prepare PP/PA6 in situ microfiber composites. After the formation of in situ microfibers, the tensile strength of the composites significantly improved from 19 MPa to 47 MPa, increasing by 147%.

In addition to adding fibers, introducing external fields during processing to form self-reinforcement structures is also a common method of polymer reinforcement. Shear field is one of the popular external fields in polymer processing. In general, polymer materials tend to form highly oriented structure (shish-kebab) under a strong shear field [28]. Therefore, some new processing technologies (such as VPIM [29], dynamic packing injection molding (DPIM) [30,31,32], loop oscillatory push–pull molding (LOPPM) [33,34,35], multi-flow vibration injection molding (MFVIM) [36,37], etc.) have been developed to provide a strong shear field, which could introduce plenty of shish-kebab structures into the polymer products, thereby significantly improving the strength and modulus of the materials. For example, Liu et al. [38] prepared simultaneously self-reinforced and self-toughened HDPE products using LOPPM. The results indicated that the tensile strength and modulus of the samples improved by about 1.8 and 1.2 times, respectively. Liu et al. [35] used LOPPM technology to prepare high-performance HDPE/ultra-high-molecular-weight polyethylene (UHMWPE) samples. The results showed that under the effect of the dynamic shear field provided by LOPPM, a large number of shish-kebab structures were introduced into the products. The tensile strength, modulus and impact strength of the materials were increased by 2.8, 4.9 and 5.8 times, respectively. Mi et al. [39] prepared iPP samples with different thicknesses of the shear layer by using MFVIM and investigated the effect of the content of the shish-kebab on the mechanical properties of iPP materials. The results showed that the content of the shish-kebab was related to the duration of the shear field; the tensile strength and modulus significantly improved with the content of the shish-kebab.

In this work, we chose PA6 to modify HDPE. This is because the mechanical strength of PA6 is much higher than that of HDPE [40,41]. Meanwhile, we applied the MFVIM technique to prepare injection-molded parts. On the one hand, we would like the PA6 dispersed phase to form in situ microfibers under the effect of a strong shear field. On the other hand, it is expected that plenty of shish-kebab and hybrid shish-kebab structures can be formed to achieve the purpose of enhancing the mechanical properties of HDPE. The results show that the content of fibers as well as (hybrid) the shish-kebab improves with the increase in vibration times (the duration of the shear field). The tensile strength and modulus of products are significantly improved, increasing by 91% and 32%, respectively.

## 2. Materials and Methods

### 2.1. Materials

HDPE (4902T) was purchased from Yangzi Petroleum Chemical Co., Ltd., Nanjing, China and the melt flow rate (MFR) is 0.3 g/10 min (190 °C/5 kg). PA6 (M2500I) was purchased from Guangdong Xinhui Meida Nylon Co., Ltd., Jiangmen, China with an MFR of 26.4 g/10 min (190 °C/2.16 kg).

### 2.2. Sample Preparation

**Figure 1:**
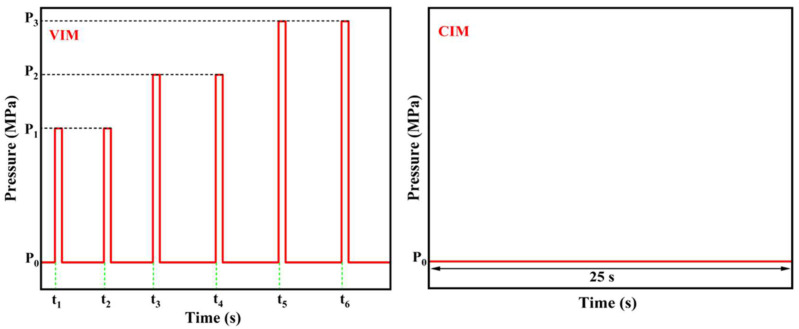
The relationship between the vibration pressure and time of VIM and CIM samples during the packing stage.

**Figure 2:**
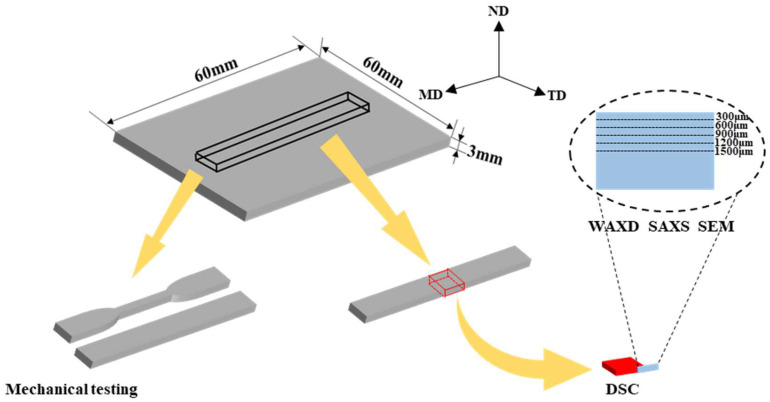
Schematic diagram of sampling method for characterizations. FD: flow direction, TD: transverse direction, ND: Normal direction.

PA6 was dried at 80 °C for 12 h under vacuum to prevent degradation during processing. Then, HDPE/PA6 pellets with a mass blending ratio of 90:10 were fully melted and mixed through a co-rotating twin-screw extruder with L/D = 40 (CPE 20 plus, Coperion, Nanjing, China). The temperatures from the hopper to the die head were between 225 and 240 °C, and the screw rotation was maintained at 140 rpm. The extruded strand was then pelletized, and the obtained blend granules were dried at 80 °C for 12 h again. In this experiment, the dried blended granules were processed into injection-molded parts by the self-developed MFVIM equipment, whose mechanism was described in detail in our previous studies [37,42]. The mold temperature was 40 °C, and the melt temperatures from the hopper to the nozzle were 180 °C, 190 °C, 210 °C, 220 °C and 220 °C, respectively. During the packing stage, vibration times were set to 2, 4 and 6, respectively. And the specific parameter settings are shown in Figure 1 and Table 1. For the purpose of comparison, conventional injection molding (CIM) samples of HDPE/PA6 blends were also prepared in this experiment. The prepared samples are denoted as CIM and VIM-x, where CIM and VIM represent the samples molded by CIM and MFVIM, while x means the vibration times. The sampling methods for various characterizations are shown in Figure 2.

### 2.3. Differential Scanning Calorimetry (DSC)

The thermal behavior and crystallinity of different samples were analyzed using a differential scanning calorimetric instrument (TA Q250, TA Instruments, New Castle, DE, USA). All measurements were performed under a nitrogen atmosphere. About 3–5 mg of the samples was heated from 40 °C to 240 °C at a heating rate of 10 °C/min. The crystallinity (X_c_) of each sample can be calculated according to the following equation:

(1) $$X_{c}=\frac{∆H_{m}}{w_{f}{∆H}_{m}^{0}}\times 100\%$$

where $∆H_{m}$ indicates the measured value of the enthalpy of melting obtained from the DSC experiment. ${∆H}_{m}^{0}$ means the enthalpy of the melting of completely crystallized PLA, which is 293 J/g [43]. $w_{f}$ represents the mass fraction of HDPE in the mixture, which is 0.9 in this work.

### 2.4. Scanning Electron Microscopy (SEM)

In order to examine the distribution and morphology of the dispersed phase in different samples, we etched the specimens by using formic acid to remove the PA6 dispersed phase. To further observe the crystal morphology, the samples were placed in a mixed acid solution for etching to remove the amorphous phase. The etched surfaces were cleaned with distilled water in an ultrasonic bath and dried afterwards. After gold sputtering treatment, the etched surfaces were examined by a field-emission scanning electron microscope (Nova Nano SEM450, FEI company, Hillsboro, OR, USA) along the MD-ND plane with an accelerating voltage of 10 kV.

### 2.5. 2D-WAXD and 2D-SAXS Measurements

Two-dimensional wide-angle X-ray diffraction (2D-WAXD) and two-dimensional small-angle X-ray scattering (2D-SAXS) measurements were carried out on the BL16B1 beamline of the Shanghai Synchrotron Radiation Facility (SSRF), Shanghai, China. The size of the rectangular beam was 0.5 × 0.8 mm^2^, and the wavelength of the light was 0.124 nm. The detector-to-sample distances for WAXD and SAXS were 88.5 mm and 1000 mm, respectively. The rectangular beam, perpendicular to the MD-ND plane in Figure 2, was moved from the upper surface to the center region of the samples and irradiated at five positions with an interval distance of 300 μm. The distances between the upper surface and each position are about 300, 600, 900, 1200 and 1500 μm, respectively.

The crystal orientation is calculated by using Herman’s orientation function, which is defined as

(2) $$f=\frac{{3<{cos}^{2}\varphi >}_{hkl}-1}{2}$$

where ${\left\langle {cos}^{2}\varphi \right\rangle }_{hkl}$ is the orientation factor, which is defined as

(3) $${cos}^{2}\varphi =\frac{\int_{0}^{\pi /2}I\left(\varphi \right)sin(\varphi ){cos}^{2}\varphi d\varphi }{\int_{0}^{\pi /2}I\left(\varphi \right)sin\varphi d\varphi }$$

where φ is the angle between the molecular chain direction and the melt flow direction, and I(φ) is the scattering intensity at angle φ. When f is equal to 0, the molecular chains are randomly arranged. When f is −0.5 or 1.0, the c axes of all the crystals are exactly perpendicular or parallel to the flow direction, respectively.

### 2.6. Mechanical Test

The mechanical strength of injection-molded products is tested using an electronic universal testing machine (68TM-10, Instron, Boston, MA, USA). According to ASTM D-638-V, the samples for the tensile test were cut into dumbbell bars with the dimensions of 65 mm × 4 mm × 3 mm (length × width of narrow part × thickness). All tests were carried out at room temperature with a cross-head speed of 50 mm/min. Five specimens of each group were tested, and the results were averaged.

A LX-D Shore Hardness Tester (HANDPI, Yueqing, China) was used to measure the surface hardness of the injection-molded parts. According to ASTM D2240, the hardness test was performed at room temperature with a specimen thickness of 6 mm. The hardness of the samples was tested at five different locations on the surface of each specimen, and the results were averaged.

## 3. Results and Discussion

### 3.1. Phase Morphology

**Figure 3:**
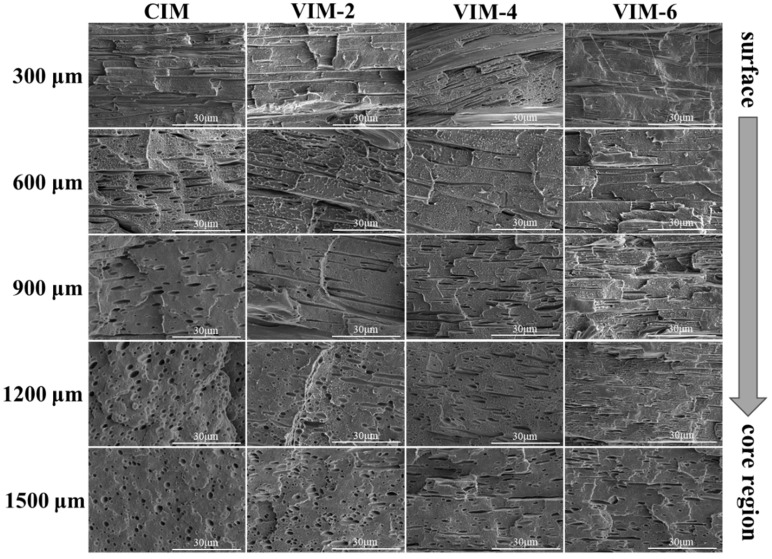
SEM micrographs of CIM, VIM-2, VIM-4 and VIM-6 at different positions with a certain distance away from the surface.

In order to investigate the morphology of the dispersed phase at different locations in the samples, the etched surfaces were observed by SEM, and the results are shown in Figure 3. For CIM, although the dispersed phase can be stretched along the flow direction due to the shear effect introduced by the filling process, it is limited around 300 μm that oriented fibers can be observed. Meanwhile, at 600 μm, the PA6 dispersed phase gradually transforms from fibers to rod-like particles with a low length–diameter (L/D) ratio. Moreover, there is a typical sea-island structure in the core region, in which the dispersed phase is mainly ellipsoidal or spherical. However, after the introduction of a strong shear field, the situation changes dramatically. For VIM-2, in situ microfibers with a large L/D ratio can be observed at both 300 μm and 600 μm. In addition, the dispersed phase can still form oriented short rods at 900 μm and 1200 μm. Meanwhile, for VIM-4 and VIM-6, highly oriented fibers can be found from 300 μm to 1200 μm. Furthermore, there are still a small number of fibers remaining at 1500 μm. From the above results, it can be concluded that in situ microfibers can be formed under the effect of a strong shear field provided by the MFVIM technology, and with the increase in vibration times, the distribution of fibers gradually expands to the core region.

### 3.2. Thermal Behavior

**Figure 4:**
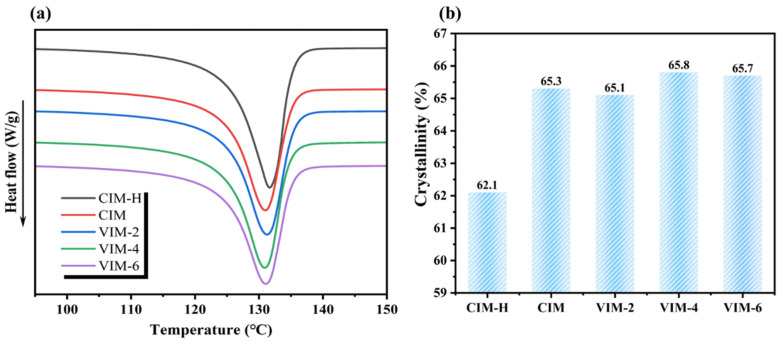
(**a**) DSC melting curves and (**b**) crystallinity of CIM-H, CIM, VIM-2, VIM-4 and VIM-6, respectively.

Figure 4a shows the DSC curves of the composites prepared under different processing conditions, and the crystallinity calculated according to the curves is displayed in Figure 4b. In order to illustrate the role of the PA6 dispersed phase in the crystallization process of the HDPE matrix, we also performed DSC analysis on CIM samples of pure HDPE (donated as CIM-H). As can be seen from the figure, the crystallinity of CIM-H is 62.1%, while the crystallinity of the HDPE/PA6 composites are all around 65.5%. The crystallinity of the HDPE matrix increases by about 3.4% after the addition of the PA6 phase. Therefore, in this experiment, the improvement in the crystallinity of the HDPE matrix is mainly due to the heterogeneous nucleation of the PA6 dispersed phase, and the difference in the morphology of the dispersed phase has little effect on the crystallinity of the matrix.

### 3.3. Crystalline Structure

**Figure 5:**
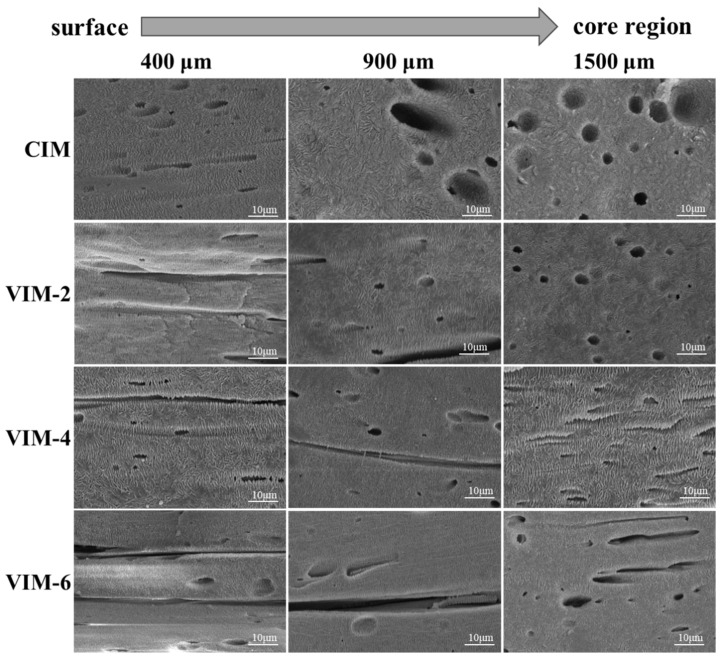
The crystalline structure of CIM, VIM-2, VIM-4 and VIM-6 at different positions with a certain distance away from the surface.

In order to obtain detailed information about the crystal structure of the samples, the amorphous phase was etched away using a mixed acid solution, and the etched surfaces were characterized by SEM. The SEM images taken at different locations of different samples are shown in Figure 5. For CIM, some lamellae oriented along the flow direction can be observed at 400 μm, while at other locations, the lamellae are randomly aligned. After the introduction of a strong shear field, although the obtained samples are all dominated by oriented structures at 400 μm and 900 μm, there is still a difference in the crystal structures. For VIM-2, some shish-kebabs can be seen at 400 μm, while the crystal is mainly the oriented lamellae at 900 μm. For VIM-4 and VIM-6, it can be found that plenty of shish-kebab and hybrid shish-kebab structures oriented along the flow direction can be observed at 400 μm and 900 μm, and the lamellae still remains oriented at the core region (1500 μm). Moreover, there are more shish-kebab and hybrid shish-kebab structures formed in VIM-6 compared to VIM-4. It indicates that the content of shish-kebabs and hybrid shish-kebabs in the samples grows with the increase in vibration times.

**Figure 6:**
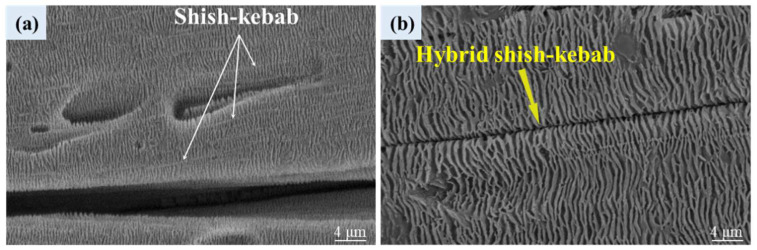
An enlarged micrograph (the magnification is 50,000×) of shish-kebab (**a**) and hybrid shish-kebab (**b**) structures in VIM-6. The distance from the surface of the samples is 900 μm.

The enlarged micrographs of shish-kebab and hybrid shish-kebab structures in VIM-6 at 900 μm are shown in Figure 6. From Figure 6a, it can be found that the molten molecular chains are highly oriented along the flow direction under the effect of a strong shear field. Moreover, during the cooling process, the HDPE lamellar grows in attachment to the oriented molecular chains to form shish-kebab structures. Meanwhile, for hybrid shish-kebab structures, it can be seen from Figure 6b that the PA6 in situ microfibers replace the oriented HDPE molecular chains as the “shish” and the oriented HDPE lamellar grows periodically on the surface of the fibers.

**Figure 7:**
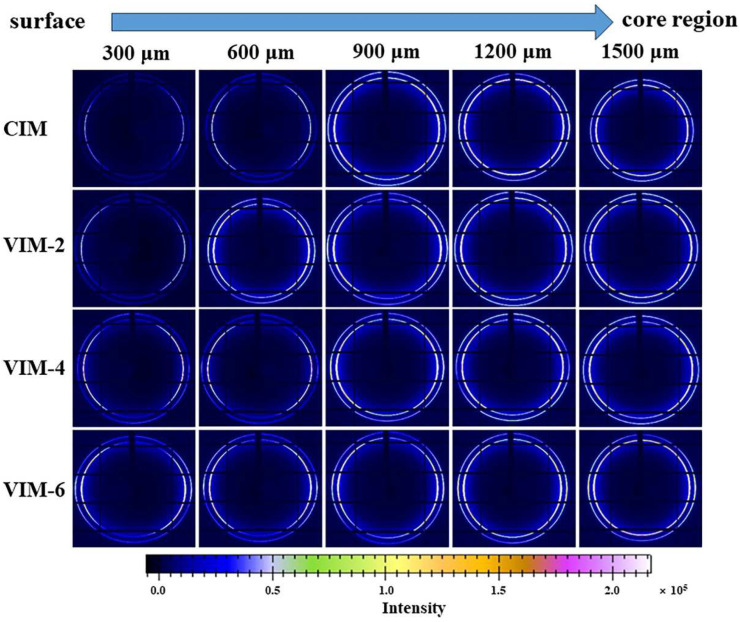
2D-WAXD patterns of CIM, VIM-2, VIM-4 and VIM-6 at different positions with a certain distance away from the surface.

The 2D-WAXD patterns of different samples at different locations are given in Figure 7. The diffraction pattern consists of two diffraction rings, where the inner one and outer one represent the (110) and (200) lattice planes of HDPE, respectively. The diffraction patterns of CIM are arc-like at 300 μm and 600 μm, indicating that there are oriented structures formed at these locations. Meanwhile, at other locations, the diffraction patterns exhibit typical isotropic diffraction rings. This suggests that during the process of CIM, the formation of oriented structures is limited in thickness due to the weak shear effect. However, for VIM-2, it displays arc-like diffraction patterns at the positions of 300 μm to 900 μm. Meanwhile, at 1200 μm and 1500 μm, the diffraction patterns are isotropic diffraction rings. In addition, the diffraction patterns all show arc-like patterns from 300 μm to 1500 μm for VIM-4 and VIM-6, indicating that the oriented structures are generated from the surface layer to the core layer in these two samples. In general, the distribution of oriented structures in the samples is consistent with the results of SEM.

**Figure 8:**
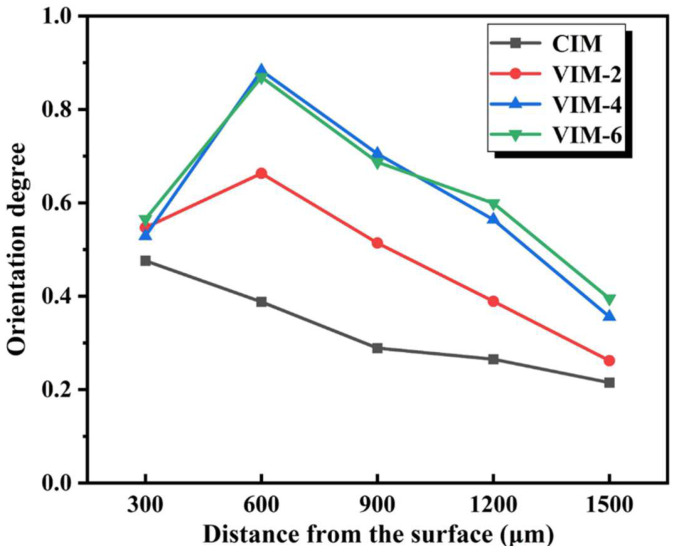
Orientation degree of different samples.

In order to quantitatively investigate the orientation degree of different layers in different samples, the calculated results of the orientation degree are summarized in Figure 8. The orientation degree of CIM is kept at 0.2–0.4 (except 300 μm) and always lower than that of the MFVIM samples. For MFVIM samples, as the distance from the surface increases, the orientation degree of all the composites rises sharply and then falls. Moreover, the orientation degree of VIM-2 is obviously lower than the other two samples, while the orientation degree of VIM-4 and VIM-6 shows little difference. This suggests that the orientation degree improves dramatically with the increase in vibration times. However, when the number of vibrations is greater than four, continuing to increase the vibration times has little effect on the improvement in the orientation degree.

**Figure 9:**
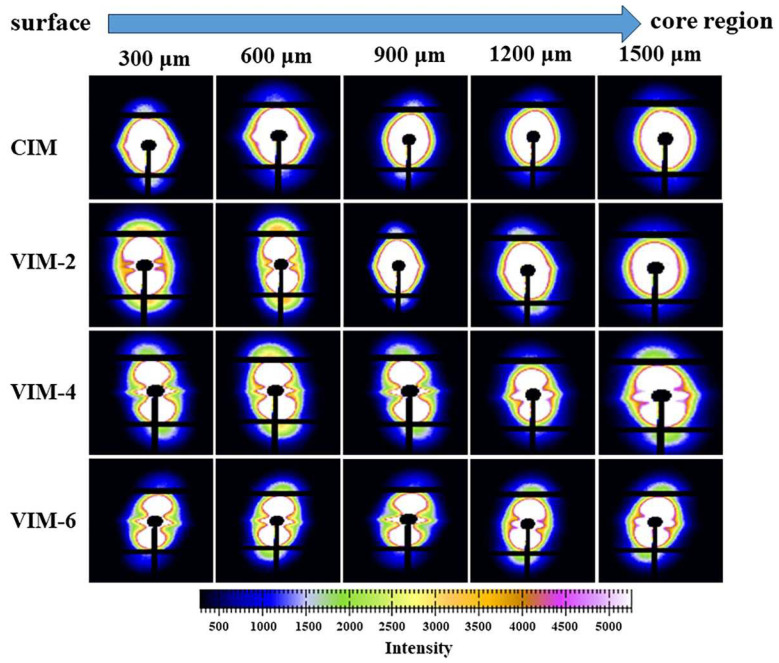
2D-SAXS patterns of CIM, VIM-2, VIM-4 and VIM-6 at different positions with a certain distance away from the surface.

To further study the difference in the crystal structure of HDPE/PA6 composites prepared by different processing conditions, the 2D-SAXS patterns of CIM, VIM-2, VIM-4 and VIM-6 samples at different positions are shown in Figure 9. For CIM, orientation signals appear in the equatorial direction of the patterns at 300 μm and 600 μm, indicating that there are oriented fibers or crystals formed in the sample at these locations. On the contrary, other positions of CIM show typical isotropic scattering patterns, meaning that both the dispersed phase and crystal at these positions are randomly distributed. As for the MFVIM samples, typical shish-kebab signals are observed at 300 μm and 600 μm in all three samples, including a “shish” signal along the equatorial direction and a “kebab” signal along the meridian direction. This suggests that under the effect of a strong shear field, the surface layer of the samples all form highly oriented shish-kebab structures. In addition, shish-kebab signals also appear at 900 μm and 1200 μm of VIM-4 and VIM-6, whereas only conventional orientation signals appear at the same positions of VIM-2. Surprisingly, weak shish-kebab signals can be observed at 1500 μm in VIM-4 and VIM-6, suggesting that small amounts of shish-kebab structures form in the core region of the two samples. Furthermore, it is evident that the shish-kebab signals at 300–900 μm are stronger with the increase in vibration times.

### 3.4. Mechanical Properties

**Figure 10:**
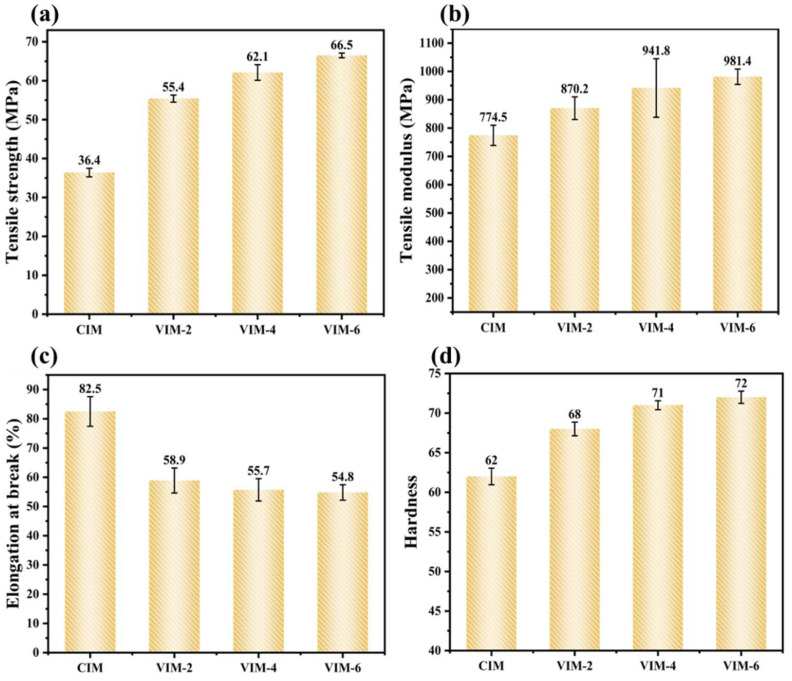
Mechanical performance of different samples prepared by different conditions: (**a**) tensile strength, (**b**) tensile modulus, (**c**) elongation at break and (**d**) hardness.

The tensile strength, tensile modulus and elongation at break of different samples are given in Figure 10a–c, respectively. To explain the role of the PA6 dispersed phase in composites for the enhancement of mechanical properties, we also performed tensile tests on CIM-H. The tensile strength, tensile modulus and elongation at break of CIM-H (not shown in the figure) are 34.8 MPa, 744.3 MPa and 162.2%, respectively. From Figure 10a,b, it can be seen that after the addition of the PA6 dispersed phase, the tensile strength and tensile modulus of CIM increase to 36.4 MPa and 774.5, respectively. The improvement is very limited, which is due to the incompatibility of the HDPE matrix with the PA6 dispersed phase, although PA6 is much stronger than HDPE [44]. However, after the introduction of a strong shear field, the tensile strength and tensile modulus of the samples improve significantly and increase with increasing vibration times. VIM-6 exhibits the maximum tensile strength and tensile modulus, which are 66.5 MPa and 981.4 MPa, respectively. Compared with CIM-H, the tensile strength and tensile modulus increase by 91% and 32%, respectively. Meanwhile, compared to CIM, they improve by 83% and 27%, respectively. The reason for the enhancement of the mechanical strength is the massive generation of in situ microfibrils, shish-kebab and hybrid shish-kebab structures under the effect of the strong shear field [8,31].

Meanwhile, for tensile toughness, it can be noticed from Figure 10c that the sample CIM possesses the maximum elongation at break. However, compared to the CIM-H sample, there is a considerable decrease in the tensile toughness of CIM after the addition of the PA6 dispersed phase, from 162.2% to 82.5%, which decreases by nearly 50%. After the introduction of the strong shear field, the elongation at break continues to decrease. But the elongation at break of all MFVIM samples remains above 50%, and the difference among them is small. It indicates that the formation of microfibers and oriented structures decreases the elongation at break of the material [35,39], while increasing vibration times has little effect on the tensile toughness. In addition, the MFVIM samples can maintain an acceptable level of toughness (>50%) while the mechanical strength is significantly improved. This suggests that products with balanced strength and toughness can be prepared through this method.

In order to investigate the ability to resist the localized plastic deformation of the material, the surface hardness of the material was tested [45]. The results are shown in Figure 10d. As can be seen from the figure, CIM has the lowest surface hardness of 62. After introducing the strong shear field, the surface hardness of the samples increases. The surface hardness of VIM-2 reaches 68, improving by 9.6% compared to CIM. Moreover, the surface hardness of the material further increases with increasing vibration times. VIM-6 possesses the maximum surface hardness of 72, which is 16% higher compared to CIM. This suggests that the formation of in situ microfibers as well as shish-kebab structures within the material facilitates the surface hardness of the HDPE material.

## 4. Conclusions

In this work, high-performance HDPE/PA6 microfibril composites were successfully fabricated by MFVIM technology. Under the effect of the strong shear field, the PA6 dispersed phase forms in situ microfibers and a large number of shish-kebab and hybrid shish-kebab structures are formed in the composites. Moreover, the distribution of microfibers gradually expands to the core region with the increase in vibration times. As a result, the mechanical performance of the products improves dramatically. The tensile strength, tensile modulus and surface hardness of VIM-6 reach up to 66.5 MPa, 981.4 MPa and 72, respectively. Compared with CIM-H, the tensile strength and tensile modulus increase by 91% and 32%, respectively. This study proposes an effective method for the fabrication of high-performance HDPE-based composites, which is of great significance for the preparation and application of HDPE products.

## Figures and Tables

**Table 1:** Processing parameters during packing stage. (P: pressure/MPa, t: start time/s).

	Packing Pressure (P_0_)	1st		2nd		3rd		4th		5th		6th	
		P_1_	t_1_	P_1_	t_2_	P_2_	t_3_	P_2_	t_4_	P_3_	t_5_	P_3_	t_6_
VIM-2	30	80	1	80	4.5								
VIM-4		80	1	80	4.5	100	9	100	13.5				
VIM-6		80	1	80	4.5	100	9	100	13.5	120	18	120	22.5

## Data Availability

Data are contained within the article.
